# Nature’s Royal Family

**Published:** 1882-05

**Authors:** George Watt


// THE DENTAL REGISTER.

VOL. XXXVI.] MAY, 1882. [No. 5.

### NATURE'S ROYAL FAMILY.

### AN ADDRESS BY GEORGE WATT.

AT THE COMMENCEMENT OF THE DENTAL COLLEGE OF THE UNIVERSITY OF MICHIGAN, MARCH 29, 1882.

ALUMNI, BRETHREN AND FRIENDS:—The first time is an overwhelming factor in the tests of human fortitude and self-possession. All things come easy by habit. Practice makes perfect. But practice implies a beginning of effort—a first time. Habit, too, speaks of test after test, and also that the thing tested, the subject of the habit, like the new boot, did not sit easy till after it had been worn a while. Ask the most experienced coquette if her cheeks did not burn and her heart flutter at the primal proposal of love's young dream. Ask the most gifted orator, the most eloquent statesman, if his maiden speech did not parch his lips and burn his throat till the necessary sip after sip suggested the idea of a windmill running by water. Ask yourselves, now that for the first time you are dubbed by a professional degree, with its legal parchment, if through all the early hours of this day you have felt easy, calm and self-possessed. Answers to all these may lead your hearts to sympathize with the embarrassment felt now and here by him who addresses you, whose wrinkled brow and frosted locks certainly testify of experience, yet not such experience that the present effort and position are not first-time trials, with their essential embarrassments, requiring a good degree of moral courage and will-power to prevent failure. True, it was once his lot to address a class, now matured practitioners of your chosen profession, but then he was a member of the faculty of the institution conferring the degrees, and hence he was addressing the familiar faces of his late pupils, and not strangers, as now. True, it was once his lot to wait, as you, and to receive from an older institution, by similar authority, the same degree for which you have successfully contended; but then he listened to the valedictory, while now he delivers it.

The period reached by you this day, in the college curriculum, is called the COMMENCEMENT. In your careers of professional lives you have attained to your commencements. And, doubtless, in looking forward to it you have slyly felt that the term is a misnomer. Commencement, indeed! You have regarded it as the close, the end, and not the beginning of your professional efforts. But were you professional men at the opening of the college term? Were you such even this morning? Verily, it is the *commencement* of your professional lives, the commencement of your lifelong battle with disease and suffering. Let it be your commencement in fulfilling the high, hopeful and honorable resolutions silently adopted by each of you for the government of your conduct and lives, as you practice the profession of your choice. And when the touch of Time has frosted your now youthful locks, and his sharp finger-tips have scraped furrows in your brows, you will recall this event and its scenes, as the world-wide wanderer looks back to the day of his departure from the parental mansion, and then, indeed, will this seem your commencement in the most rigid interpretation of the word, and as the wanderer clings to and cherishes the parental gifts and parting tokens, then will you honor and prize the parting tokens given you this day by your *alma mater*.

But what shall I say? Your time must not be wasted by mere idle words. But no word of mine, now and here, can better fit you for the professional battle before you. I must take for granted that you are guided by the examples of your teachers, as you have been qualified by their instruction, while by the parchments in your possession I know you have been endorsed by their judgment--that weighed in the balances of impartial, exhaustive examinations you are not found wanting, but have reached "unto the measure of the stature of the fullness of" professional man.

hood, and are therefore my equals, ready and able to confer with me as to our mutual interests in professional science, and are no longer babes or children to be fed on mental pabulum digested ready for your intellectual nutrition. But found on the same professional level, members, indeed, of the same family, let us join in fireside discourse worthy of the family to which we belong. Let us talk of a topic that will interest all the members of the family.

As floating gossamers, wafted by the breezes of the universe, shall we discuss our fellow molecules? As fragmentary matter, shall we explore our structure and investigate the question of the elements used in our formation? Shall we tarry to inquire of what we are, and with what we are? Shall we survey all within, and look at all without, that we may know ourselves and our environments? No! no! Let us not fence more land than we can till. Let us not overtask, lest we be tempted to overstep. These suggestions all imply work, while age, on my part, hints at quiet and rest, and, on your part, months of toil, in the companionship of lamps by night and lectures by day, call for relaxation, if not amusement. But the tyrant fashion or habit expects us to spend this hour together; and how shall we spend it? I am an invited guest to a meeting for friendship. You would not put me to work. You are the heroes of the meeting, the youngest sons and daughters, and therefore the pets of your *alma mater*, bidding her this day farewell, and going forth with her blessing, and thus, as you are trimmed out in your holiday suits of mental attire, we must not exact toil of you. But the grim tyrant, habit, grudging time squandered, demands that we do something; but can we not steal a march on his morose highness, and amuse ourselves while pretending to work by playfully studying pleasant scenes and incidents in the biography of the royal family of the Kingdom of matter? Thus:

In the long ago, when Time was a babe, before the cock-crowing for Creation's dawn; about the time the morning stars sang their first concert, and the sons of God shouted an encore for joy, there was a royal element, a grand, princely old entity, who from the dignity of his character and the mightiness of his power, his influence over his comrades, and various other royal attributes, was recognized by all of materiality as Lord Oxygen. His throne is on the storm-cloud, and the forked lightning is his scepter; "his pavilion round about him are dark waters and thick clouds of the skies; he flies upon the wings of the wind;" hailstones are his messengers, and coals of fire are kindled by his breathings, as channels of waters are seen in his pathways. The world's foundations were laid by his hand, to perish in the great day at the blast of the breath of his nostrils. The earth quakes and trembles in the assertion of his power, and the hills move and are shaken when he is wroth. He steps on the mountains and they burst forth in volcanoes. He walks over cities, and they are carried away in conflagrations, or crumbled into dust. And at all times his energy and industry are co-ordinate with his power. Other monarchs rest after they have reached their thrones, and their titles are recognized; but success seems only to drive him to deeds more daring and desperate. And nothing escapes him, for he seems almost omnipresent. Climb to the highest peak of the loftiest mountain range, and he is still above and around you. Dig into the deepest recesses of mundane mysteries, and his works testify he has been there before you. Fly to the ends of the earth to escape his presence, and he will laugh as he shows you that not your own efforts, but his assistance has carried you thither. Drown yourself in the ocean's depths, and he will lay you gently down on his own quiet bed, and shroud your pale corpse with winding sheets of his own weaving. To us he is practically omnipresent; for he goes whither we go, abides where we abide, and we cannot away with him. Ask not, then, where is this mighty king of material elements. Ask, rather, where is he not, since he is high above the mountain's crag, and far below the ocean's bottom; since he carries the eagle in his loftiest flights, and accompanies the miner deep into the bowels of the earth, and is there even before him. Shall we ask the way to the palace of the king who rides on the swift wings of the wind and rejoices in the society of the storm-cloud and the tornado, playthings of his own creative fancy? Would you meet him in the middle of the mountain, seek him in the solid rock, or look for him in all things that live? Go, then, and your quest shall meet with its full reward.

But hold! Besides his practical omnipresence, with god-like attribute, he is quite invisible; and how shall we recognize him? "Seeing is believing;" but you cannot see him. You may meet him in the highways and byways, and his presence is unappreciated. You may lie folded in the embrace of his mighty arms, but you may not look on the majesty of his countenance; for eye of mortal may not see even his back parts. His presence is felt, not seen. He is beheld in his works; but form and feature remain unseen.

If seen only in his works, it becomes us to inquire what they are, and where are they? But tongue must fail, and pen fall short, in announcing and recording their catalogue. He forms the ocean and the land, controls the atmosphere, supervises all changes on the face of the earth. Ever busy, he forces his kindred elements to obey his absolute will, and toil with him in his manifold labors of benificence or woe. He causes one to form the dew and the raindrop, the snowflake and the frost-crystal, the icicle and the glacier, the cascade and the cataract, the fountain and the rill, the river and the ocean, the storm cloud and the rainbow, and the crimson curtain that veils the setting sun—

> Ere jocund day subdued departs,
> And somber night lights up his lamps.

With another element he blends to form the balmy atmosphere, thus giving breath—

> "Enough to satisfy the needs
> Of every thing that lives."

He fans the brow of the bashful maid till he bleaches it to marble; yet, with the same breath, he paints her cheeks to the rose's hue. He floats the gossamer, fills the sails of the merchant's ships, and carries aloft the school-boy's kite. He sets forth his wrath in the whirlwind, his smiles in the zephyr, and his lullaby song in the southern breeze. When greatly enraged he carries the pestilence in his bosom, and sows it broadcast before him as he goes breathing out "threatenings and slaughter" against all found in his pathway; but when pacified he regales us with the perfumes of Araby the blest. He causes the "spikenard to send forth the smell thereof, with the fragrance of a cluster of camphire in the vineyards of Engedi, whence he causeth the vines with the tender grape to give a good smell, as he cometh out of the wilderness perfumed with myrrh and frankincense." The north wind awakes, and the south wind comes at his bidding, to blow upon the gardens of spices which he has cultivated for his own delights. He, too, carries the rain clouds, and assorts and sifts the snowflakes. He holds up the vapors that form the refreshing dews and the beautiful frost-crystals; and he hangs up the black cloud as a background to the bow of promise, the token and seal of the earth's safety. But time would fail to tell of a tithe of his ways and deeds while partner with this one element trained to his service.

He teaches another to form the rocks that we may build houses for protection and castles for safety, while he forms, assorts and sifts the sands of the seashore for pastime. He burns a bright metal to lime, then blots the diamond from our sight, giving in its place a deadly poison, which is held from harm by the ready lime, thus forming the solid marble that beauty may be perpetuated by the sculptor's chisel. He wipes out want by supply, through ships wafted and carried to desired havens, or he forges the lightning train, and on tracks of his own construction he defies resistance, mocks at time and laughs at distance, making man ubiquitous. But these only when he is at leisure. If pressed for time he commands the lightnings and sends them on servile errands over lands and under seas, outstripping time along the wiry ways he has laid for their guidance.

Should not such an element be patronized and pacified? for none have hardened themselves against him and have prospered. Iron and brass are but brittleness with him, and weapons of steel he grinds to powder. Silver is blackness in his touch, and gold becomes fine dust before him. The leaves of the forest wither at his touch, and nature shrinks from his blasting energies. Think of the destructions and desolations in his career of ruin! Nations have been blotted out by his breath. Great Babylon and the

"eternal city" have fallen by his hand; and while the winds wail a requium over the dead past, he raises a shout of triumph in the gale, and rushes on to new conquests. He forges the thunderbolts of war, devours the widow's bread to form the demon drink that fills the world with woe, and changes men to devils. He is the pitiless executor of the sentence, "Dust thou art, and unto dust shalt thou return."

With all these before us, like sages who sacrifice to devils, and savages who worship the storm-king, shall we fall at his feet and do reverence to this mighty element which will one day crumble us to dust? Nay, we must inquire further; for we assimilate that which we worship. Let us then turn from these traits and think of his goodness ; and possibly here we shall find something befitting our homage. At being's dawn he breathed into our nostrils the breath of life, and he breathes new life into us still every second, furnishes us food and drink, comfort and safety, amid luxuries and blessings beyond the fancied stories of fabled dreams. He smiles through all our senses, he paints the flowers and dyes the gay green robe of field and forest. And even "when chill November's surly blast," with wailing winds, announces the fading of the foliage and the death of the flowers, he bedecks the forests with death-robes of delight and beauty till "even Solomon, in all his glory," was not so gorgeously arrayed. He paints the leaves with crimson, and gold, and purple, and scarlet, till even the fastidious south wind accepts them as substitutes, and no more

> "* * * Searching for the flowers
> Whose fragrance late he bore."

And daily and hourly he changes the scenes, displaying new pictures of glory and beauty till the eye wearies with the variety.

And now, must an element so noble, grand and powerful, so arbitrary yet so cordial, must he muse in solitary grandeur? Must he pine in the solitary loneliness of isolation? Nay, verily! Read the family record of the royal household of the Kingdom of Nature. There we learn that He who sits on the throne of the universe, whose is the dominion, and the power, and the glory, thought it not good that an element so lordly should be alone; hence, "He spake and it was done," and thus a helpmeet for him was provided and a beautiful bride sprung up by his side, and their marriage was sealed by the lightning's flash, and announced by the thunder's roar, and we are told in the record of creation, "that darkness was upon the face of the deep, and the Spirit of God moved upon the face of the waters." Thus was it decreed by the Mighty One that those royal atoms should be no more twain, but one molecule. Thus, in the annals of science, have we recorded the formation of the first family in the great kingdom of the material universe. As the lightnings have revealed and the thunders announced the marriage of the stately, grim Lord Oxygen, we must announce the bride as the quiet, meek and beautiful Lady Hydrogen. And now, would you worship at the shrine of antiquity and revere first families, here is something worthy of your devotion, found in a match of affinity, a case of pure love at first sight, a union so close that for thousands of years these royal atoms were regarded as if even by nature not twain, but as they really are, one.

But what of the bride? Who? and of what household and lineage? Whence comes she? Hold! How can I introduce her? She will not sit for her likeness, nor even unveil. As a modest wife she glories in her lordly husband, but shrinks timidly from all display. Yet is she known by her works, more truly than is her royal husband. In deeds of benificence she is above all. "She stretcheth out her hands to the poor; yea, she reacheth forth her hands to the needy." While many have done virtuously, yet she excels them all. Ask the forest the secret of its growth and beauty, and it refers you to the good Lady Hydrogen. The flowers display their bright hues and fragrance as gifts from the bride of Lord Oxygen. You see her pencilings in the rainbow, and her art touches in the rose. All the elements rejoice in their queen, and delight to serve and honor her. And why not? Why not even we adore and worship such brilliance, and beauty, and gentleness, and purity, and grandeur, and tenderness, and faithfulness, and industry, and energy? Shall the queen of science, with all these attributes, be viewed with indifference? Shall we become sentimental over the beauty of Helen? or enthuse before the statue of Venus, and not be filled with rapture in the presence of the queen of all beauty, as pure as the angels, the seraphic bride of Lord Oxygen? "As pure as the angels!"—as purity itself. The touch of filth cannot defile her, nor the slanderous tongue defame her, even though the poison of asps were under the lips that aid its utterance.

"Beauty is vain," and oft a temptation, but never when based on such purity as her's. Tempted by the fury of passion, those elemental bandits—Sulphur, Chlorine and Phosphorus—kidnap and carry her off, yet they find in her beauty charms not for them. The grandeur of countenance displayed in her constancy appals them, and protects her from outrage, and where they expected riotous fullness of joy, because of her presence, all is acrid and sour; and when her royal husband comes to her rescue and consumes them with the breath of his mouth, spotless and pure as the virgin immaculate, she flies to his protecting embrace "with joy as a new-made bride," "and the heart of her husband doth safely trust in her."

But of what value are all her grandeur, queenliness, gentleness and beauty, in comparison with her goodness? She blesses, not for applause, but for sake of blessing. So airy, light and gay, yet so gentle withal, we would expect her to play with the gossamer, romp with the butterfly, or sport with the blue ether, beyond the clouds, above the sky and among the stars, to join the meteors in their merry chase after the comets, to play "hide and seek" in the milky way, and bespangle her toilet with star-dust; but not so with our lady queen, for all this were useless; and had she the strength of iron and brass, the gravity of lead, and the caustic energy of chlorine, she could undertake and accomplish no more valorous deeds. Marvelous is her power, and wonderful are her works, yet all is done by love. So gentle is her reign that her subjects become her willing slaves and forget their servitude in her sisterly kindness. Her force of character is best illustrated by her influence over her royal husband. His passions are subdued by her affection till he is as gentle as the lamb. Alone, the forest withers in the blast of his fiery breath, and the face of Nature blanches at the touch of his finger. Pacified by her presence, he waters the earth and clothes it with a carpet of green, and bedecks it with flowers more beautiful than the gems that bedecks the diadems of royalty. Solitary, he burns cities to ashes; but when present, he gratifies her by drowning the conflagration. Without her society he wrecks the stately ship; yet. encouraged by her smiles, he floats it to the desired haven. His best deeds are done through her inspiration, and often he is helpless without her.

In reference to human society, it has been quaintly said that the husband and wife are one, and that one is the husband; but not so with this royal pair, who govern the United States of the Elements, as she is ever and always the recognized equal of the husband of her choice.

Feeling, as we all do, that "blood tells," when such a marriage occurs in royalty all the kingdom takes interest in the question of perpetuation. When the crisis—the hour of trial has been reached and passed, and the glad news has been whispered that "all is as well as can be expected"—in short, when an earthly queen has brought forth a man-child, the heir apparent, the prince imperial, the first-born of royalty, the future monarch of the kingdom, sympathy for the tried and triumphant mother is for the time forgotten, for joy that a man is born into the world, and shouts of triumph commingle with the clang of martial music, and subjects join with one accord in demonstrations of welcome to the new-born king, prisoners are pardoned, crimes are condoned, the guilty go free, new friendships are formed, and all join in jubilee.

In like manner, and more so, was the prince imperial, the heir apparent, the first-born and only son of the royal family of the kingdom of matter, welcomed and announced. The lightnings lit up the royal castle from foundation to turret, the thunders roared to reverberate the glad tidings from west to east, from south to north, causing the swift-winged winds to quiver with delight in their privileges as carriers of the welcome news; the meteors, joyful in their glee, darted across the sky, along the milky way, to tell the comets; the flowers sprang up on every side to embellish and bedeck the scene; the grasses made his swaddling clothes of gayest green; the roses spread o'er him their richest perfume; the violets smiled blue into his still bluer eyes; the peachblows winked at his parting lips, and the peaches copied his blushing cheeks; the dewdrops glittered because he was born; the eyes of the frost-crystal sparkled as they watched him with delight; the cascade laughed and the rill sang lullaby because he had come; the waves welcomed him as they rolled, and the river rollicked with delight as it bore him on its tide; the clouds shaded him from the scorching beams of noonday, and the mists of the morning veiled his eyes from the piercing rays of the rising sun; heaven smiled through the brightness of its firmament at the day's departure, and held out its rainbow as a token of peace; earth drank in blessings of delight as they were showered upon her, and Nature rejoiced with joy unspeakable, over the arrival of her royal prince.

How sad that we so often see the son inherit the parental estates and titles, with few or none of the parental excellences! But not so with our hero prince. He has all the good traits of both his most excellent parents. Their works have become his, and he is faithful to his inheritance. A record of his deeds and doings would be but a rehearsal of theirs. In grandeur, and power, and majesty, and skill, and beauty, and goodness, he is their equal and counterpart, a worthy son of worthy parentage.

Proud of his ancestry, heritage, and position? Nay, verily; for he toils like a slave, with the industry of thrift personified,—robes of royalty laid aside, never idle, and always going about to do good. He often disguises his form and features that he may have increased opportunities for usefulness over those possible for a recognized prince. So humble is he that he almost totally discards his titles. Seldom does he tolerate his real name and title, THE PRINCE IMPERIAL HYDRIC OXIDE. We read of royalty in disguise sojourning in the cottage of the peasant; but we never find the peasantry less respectful and obsequious after rank and title have been discovered. They revere the royal friend all the more for his social excellence, which they could not have discovered and enjoyed had they known the real rank and title. And will you regard him with less favor when you learn that your old familiar friend, WATER, your companion and playfellow, is none other than His Serene Highness, HYDRIC OXIDE, the Royal Prince of the Material Universe, the first born of Lord Oxygen and his good wife, Lady Hydrogen?

But if his titles and estates were all dropped, and he were only water, as you found him in disguise, is he not everywhere, and in all respects, "a thing of beauty and a joy forever?" Think of him as he glitters in the dewdrop, sparkles in the rain, shines in the frost-crystal, gambols in the snowflake, glistens in the icicle. See his smile in the cascade, and hear his laughter in the cataract. Listen to his music as he soothes us to rest by

> "That melody of Nature,
> That subdued, subduing strain,
> Which is played upon the shingles
> By the patter of the rain."

He, and he only, can revive and refresh the weary wayfarer, as through thirst and heat he has fainted on the sandy desert. The gold of Ophir is then but dross, and the diamond but condensed charcoal; but our plain hero, water, is he who strengthens, refreshes, revives, invigorates, and purifies. His blessings render rich and add no sorrow. Demons have no delight in him, and never rejoice in his ministrations. He never prepares the murderer for his crime, nor gives strength to the reveler for his midnight debauch. No ghosts of murdered innocents awake from their slumbers to curse the cup he filled for them, or the fountain he provided for their refreshment. Reason is never dethroned through his inspiration; nor are prisons crowded with his victims while courts of justice are kept busy with their crimes. His nobility is in his nature. His excellency of character is his own.

And now, my young brethren, if you will each, at leisure, carefully study the nature, formation, distribution, locations, offices, functions, characteristics, and properties of *water*—study all these, both in their physical and chemical aspects—you will find a field for mental exercise worthy of your best efforts, and you will reach the conclusion that your plain companion, water, is worthy of his title, His Royal Highness, Hydric Oxide; worthy of all the eulogy I have tried to bestow on him, worthy of all the commendation you can give him.

